# Opioid Overdose Outbreak — West Virginia, August 2016

**DOI:** 10.15585/mmwr.mm6637a3

**Published:** 2017-09-22

**Authors:** Joel Massey, Michael Kilkenny, Samantha Batdorf, Sarah K. Sanders, Debra Ellison, John Halpin, R. Matthew Gladden, Danae Bixler, Loretta Haddy, Rahul Gupta

**Affiliations:** ^1^Epidemic Intelligence Service, Division of Scientific Education and Professional Development, CDC; ^2^Bureau for Public Health, West Virginia Department of Health and Human Resources; ^3^Cabell-Huntington Health Department, West Virginia; ^4^Division of Unintentional Injury Prevention, National Center for Injury Prevention and Control, CDC.

On August 15, 2016, the Mayor’s Office of Drug Control Policy in Huntington, West Virginia, notified the Cabell-Huntington Health Department (CHHD) of multiple calls regarding opioid overdose received by the emergency medical system (EMS) during 3 p.m.–8 p.m. that day. A public health investigation and response conducted by the West Virginia Bureau for Public Health (BPH) and CHHD identified 20 opioid overdose cases within a 53-hour period in Cabell County; all cases included emergency department (ED) encounters. EMS personnel, other first responders, and ED providers administered the opioid antidote naloxone to 16 (80%) patients, six of whom were administered multiple doses, suggesting exposure to a highly potent opioid. No patients received referral for recovery support services. In addition to the public health investigation, a public safety investigation was conducted; comprehensive opioid toxicology testing of clinical specimens identified the synthetic opioid fentanyl* and novel fentanyl analogs, including carfentanil,^†^ which had been used by patients who overdosed in Huntington. Results of these two investigations highlight the importance of collaboration between public health and public safety agencies to provide in-depth surveillance data from opioid overdose outbreaks that involve high-potency fentanyl analogs. These data facilitated a public health response through increased awareness of powerful opioid substances requiring multiple naloxone doses for reversal, and improved patient linkage to recovery support services and a harm reduction program from the ED after opioid overdose.

## Public Health Investigation

**FIGURE 1:**
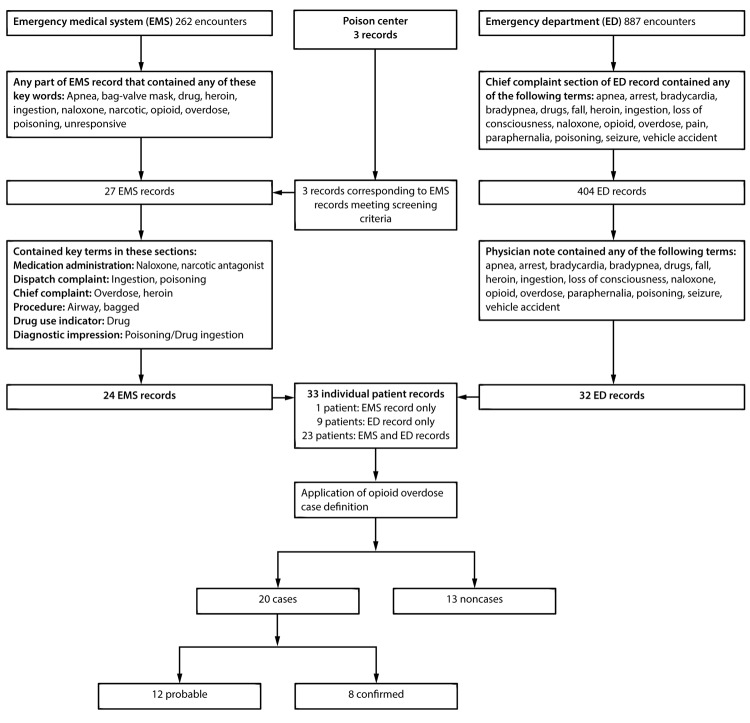
Case identification algorithm used for an opioid overdose outbreak investigation*^,†^ — Cabell County, West Virginia, August 14–16, 2016 * To identify cases, investigators collected Cabell County EMS records and records from the two Cabell County EDs for the 53-hour period from 3 p.m. on August 14, 2016, to 8 p.m. on August 16, 2016, (24 hours before and 24 hours after the 5-hour period of increased drug overdose EMS calls on August 15). ^†^ A probable case of opioid overdose was defined as 1) clinical suspicion of opioid exposure (documented by patient mention of drug use, observed drug paraphernalia, naloxone administration, or ED diagnosis of drug poisoning or drug use) and 2) one or more clinical signs of central nervous system depression (bradypnea, apnea, altered consciousness, or miosis) in a person identified through EMS or ED records. Confirmed opioid overdose cases met the probable case definition and had a positive toxicology screening result for any drug of abuse.

On August 18, 2016, CHHD requested assistance from BPH to investigate the opioid overdose outbreak in Cabell County and conducted a retrospective public health investigation to characterize the outbreak and improve public health response. Investigators collected data from multiple stakeholders, including public safety (law enforcement and fire department personnel) and health care facilities and created case-finding methods and case definitions. To identify cases, investigators collected Cabell County EMS records and records from the two Cabell County EDs covering a 53-hour period from 3 p.m. on August 14, 2016, to 8 p.m. on August 16, 2016, (24 hours before and 24 hours after the 5-hour period of increased drug overdose EMS calls on August 15). Investigators also collected West Virginia Poison Center records of prehospital naloxone administration by Cabell County public safety personnel. Investigators screened, identified, and selected records related to an opioid overdose using key terms and applied the case definition to records from the study period, using a case identification algorithm (Figure 1). Demographic information, rescue and resuscitation measures, medical history, clinical findings, and ED disposition were abstracted from all record sources and analyzed.

A probable case of opioid overdose was defined as 1) clinical suspicion of opioid exposure (documented by patient mention of drug use, observed drug paraphernalia, naloxone administration, or ED diagnosis of drug poisoning or drug use) and 2) one or more clinical signs of central nervous system depression (e.g., bradypnea, apnea, altered consciousness, or miosis) in a person identified through EMS or ED records, from 3 p.m. August 14 through 8 p.m. August 16. Confirmed opioid overdose cases met the probable case definition and had a positive toxicology screening^§^ result for any drug of abuse. A positive toxicology result for any drug of abuse was used to confirm cases because persons who abuse opioids might use multiple drugs, including nonopioids (*1*), and available clinical toxicology screening tests do not detect fentanyl or fentanyl analogs. Public health investigators did not have access to in-state confirmatory testing for fentanyl and fentanyl analogs.^¶^

**FIGURE 2:**
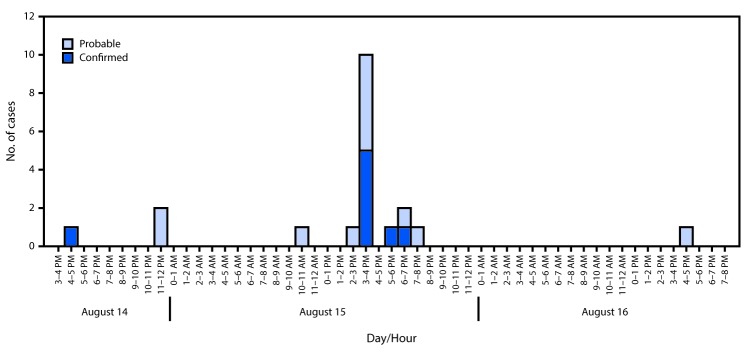
Number of probable (n = 12) and confirmed (n = 8) opioid overdose cases per hour of day — Cabell County, West Virginia, August 14–16, 2016* * As a result of the public safety investigation, carfentanil and furanylfentanyl were identified in March 2017 among patients who had been evaluated on August 15, 2016, during 3 p.m.–4 p.m. and 5 p.m.–6 p.m.

Twenty patients met the opioid overdose case definition; 12 patients had probable cases and eight had confirmed cases. Patients aged 26–35 years accounted for 50% of cases. Location of first responder contact with 17 (85%) patients was within the city of Huntington; 14 (82%) of these contacts occurred during 3 p.m.–8 p.m. on August 15 (Figure 2). All patients had ED encounters during the study period; 18 (90%) arrived by EMS. The most commonly reported clinical signs were altered consciousness (13; 65%) and respiratory failure (11; 55%). Fourteen patients (70%) reported using heroin immediately before being evaluated in the ED. Sixteen (80%) patients received naloxone; among these patients, 12 received naloxone only in the prehospital setting, two received naloxone during both prehospital and ED encounters, and two received naloxone only in the ED. Six patients received multiple naloxone doses. Among eight (40%) patients who had toxicology screenings, opioids were detected in six, and more than one substance was identified in five (Table). Twelve (60%) patients left the ED against medical advice before discharge. All 20 patients survived, although no referrals for recovery support services, including treatment of substance use disorder, opioid addiction, opioid withdrawal, or harm reduction services (e.g., naloxone prescribing or safe injection education) were documented.

**TABLE:** Demographic information, naloxone administration, toxicology results, and reported drug used for 20 persons with confirmed (n = 8) or probable (n = 12) opioid overdose — Cabell County, West Virginia, August 14–16, 2016

Patient	Age group (yrs)	Sex	Naloxone dose (administration route)	No. of naloxone doses	Total naloxone dose	Reported drug used	ED toxicology (public health investigation)	Opioid confirmation (public safety investigation)
A*	18–25	F	2 mg (IN)	1	2 mg	Heroin, crack	Cocaine	NP
B	18–25	F	0.4 mg (IV)	2	0.8 mg	NR	NP	NP
C*	18–25	F	NA	0	NA	NR	Opioid, benzodiazepine	NP
D	26–35	M	2 mg (IN)	1	2 mg	Heroin, marijuana	NP	NP
E*	26–35	F	0.4 mg (IM)	1	0.4 mg	Heroin	Opioid, cannabinoid	Carfentanil, furanylfentanyl
F*	26–35	F	2 mg (IN), 2 mg (IV)	2	4 mg	Heroin	Opioid, cocaine, cannabinoid	Fentanyl^†^
G	26–35	F	NA	0	NA	Heroin	NP	NP
H	26–35	M	2 mg (IV)	1	2 mg	NR	NP	NP
I*	26–35	F	NA	0	NA	Heroin	Opioid, cocaine, benzodiazepine	NP
J*	26–35	F	NA	0	NA	Heroin	Opioid, cocaine, cannabinoid	Carfentanil, fentanyl, furanylfentanyl
K	26–35	M	0.4 mg (IV)	1	0.4 mg	Heroin	NP	NP
L	26–35	M	0.4 mg (IV), 2 mg (IV)	2	2.4 mg	Heroin	NP	NP
M	26–35	M	0.4 mg (IV)	3	1.2 mg	NR	NP	NP
N	36–45	M	0.4 mg (IM)	1	0.4 mg	Heroin	NP	NP
O	36–45	F	2 mg (IN)	1	2 mg	Heroin	NP	NP
P	36–45	M	NR	NR	NR	NR	NP	NP
Q	46–60	M	2 mg (IV)	1	2 mg	NR	NP	NP
R*	46–60	M	0.4 mg (IV)	5	2 mg	Heroin	Cocaine	NP
S*	46–60	M	0.4 mg (IV)	5	2 mg	Heroin	Opioid	Carfentanil, fentanyl, furanylfentanyl
T	46–60	M	2 mg (IN)	1	2 mg	Heroin	NP	NP

**Abbreviations:** ED = emergency department; F = female; IM = intramuscular; IN = intranasal; IV = intravenous; M = male; NA = not applicable; NP = not performed; NR = not recorded.

* Confirmed case.

^†^ Inadequate specimen volume for fentanyl analog testing.

## Public Safety Investigation

Public safety officials conducted a separate investigation of this opioid overdose outbreak in conjunction with legal proceedings; this investigation included comprehensive opioid toxicology testing of clinical specimens obtained from the treating EDs. In October 2016, the BPH Office of the Chief Medical Examiner (OCME) and the Drug Enforcement Administration confirmed the first carfentanil-related death in Cabell County, which occurred within days of the August 15, 2016 outbreak (*2*). Results of the comprehensive opioid testing were released to CHHD on March 23, 2017, and four patients’ specimens from the public safety investigation were matched to specimens from the public health investigation cases, three of which were positive for carfentanil and furanylfentanyl. The fourth specimen was positive for fentanyl only, with insufficient specimen volume for fentanyl analog testing (Table). On April 17, 2017, the U.S. Attorney’s Office released a statement that an Akron, Ohio, resident had been convicted of heroin, fentanyl, and carfentanil distribution in Huntington, West Virginia, on the afternoon of August 15, 2016 (*3*). OCME reported that fentanyl was involved in a number of opioid overdose deaths in Cabell County during several weeks preceding the mid-August opioid overdose outbreak.**

## Public Health Response

In October 2016, BPH released a health advisory to medical providers, first responders, and public safety personnel, notifying them of carfentanil emergence in the illicit opioid supply in West Virginia, the danger of carfentanil exposure, and the role of multiple-dose naloxone administration after exposure (*2*). Fentanyl testing of decedent specimens became available in-state through OCME in October 2016. Public health stakeholders increased naloxone distribution among first responders to meet the potential need for multiple-dose resuscitation. After receiving public health investigation findings that no opioid overdose patients who met the case definition had been referred for substance use disorder treatment, CHHD and local ED staff members improved referral protocols for overdose care and recovery support services. Local EDs coordinated with substance use–disorder treatment facilities to pilot multidisciplinary response teams that follow up with patients who experience opioid overdose and ensure linkage to care availability after ED encounters (e.g., direct connection to CHHD harm reduction program staff members).

## Discussion

This report describes a nonfatal outbreak of opioid overdoses in Cabell County, West Virginia, that heralded the emergence of two powerful fentanyl analogs, carfentanil and furanylfentanyl, in the local illicit drug supply. Public health investigation revealed a narrow clustering of the majority of cases in place and time, along with requirement for multiple-dose naloxone for resuscitation, suggesting that a point-source opioid overdose outbreak involving a high-potency opioid had occurred. However, fentanyl analog screening was unavailable in EDs at the time, and therefore, comprehensive toxicology was not accessible to public health investigators. Comprehensive opioid testing is often a component of public safety investigations, and a concurrent public safety investigation subsequently identified fentanyl analogs used by three patients. Medical examiner data obtained during the outbreak period demonstrated the emergence of carfentanil among opioid overdose decedents in Cabell County during this period, providing further evidence of the debut of carfentanil among heroin users in Cabell County and its role as a cause of this outbreak. Epidemiologic evidence and public safety investigation findings were consistent with the U.S. Attorney’s Office report (*3*).

Opioid overdose outbreak preparedness requires the cooperation of public health and public safety officials to effectively investigate and characterize the scope and nature of an outbreak. Although polysubstance use was identified by ED toxicology screening in five of the eight confirmed cases, most patients who experienced overdose reported using heroin only, and none reported using a synthetic opioid. Therefore, comprehensive toxicology testing for fentanyl, fentanyl analogs, and other newly emerging psychoactive substances might be important when conducting overdose outbreak investigations. Development and implementation of opioid overdose surveillance standards, comprehensive testing capabilities, and overdose outbreak investigation tools are needed to improve rapid identification of local illicit opioid supply changes and facilitate targeted and coordinated public health and public safety response and prevention measures. In New Haven, Connecticut, a fentanyl-related overdose investigation demonstrated that collaboration between public health, health care facilities, and public safety departments improves resuscitation preparedness efforts after an opioid overdose outbreak (*4*). However, continuum of care for patients involved in an opioid overdose outbreak should not stop at the point of resuscitation. Initiating treatment for opioid use disorder in the ED has been shown to significantly increase patient engagement^††^ in addiction treatment (*5*). ED encounters during this outbreak represent missed opportunities to link persons with a nonfatal overdose to substance use–disorder treatment initiation and ongoing care.

Surveillance for clusters of opioid overdose at the local level is increasingly important because of the rapidly changing nature of the opioid epidemic in recent years as communities witness emergence of synthetic opioids among fatal and nonfatal cases of opioid overdose (*4*,*6*). Comprehensive testing for synthetic opioids is not routinely included in ED toxicology screenings, although it is often available to public safety investigators (*6*,*7*). Cooperation between public health and public safety officials during overdose outbreak investigations could facilitate timely messaging to inform medical providers and public health and public safety personnel regarding emerging drug threats.

**Summary**

**What is already known about this topic?**

Opioid overdose is a growing health threat in the United States; CDC issued a health advisory to health departments, health care providers, first responders, and medical examiners about the introduction of high-potency synthetic opioids into the illicit opioid supply, causing outbreaks of opioid overdose and overdose-related deaths. Patient administration of the opioid antidote naloxone during an opioid overdose outbreak can save lives; however, little is known about follow-up care after resuscitation of patients who experience overdose during an outbreak.

**What is added by this report?**

An investigation of a nonfatal opioid overdose outbreak that occurred in Huntington, West Virginia, on August 15, 2016, identified 20 cases during a 53-hour period (14 overdoses occurred within 5 hours) and provided evidence that a novel, high-potency synthetic opioid was introduced into a community of persons who use illicit opioids. None of the opioid overdose patients who met case criteria received referral for substance use disorder treatment or harm reduction services.

**What are the implications for public health practice?**

Local surveillance of opioid overdose that includes investigation of overdose outbreaks produces data that can direct public health response to the opioid overdose epidemic. Development of public health and public safety partnerships for substance identification, and of strategies to link overdose patients to recovery support services at the point of resuscitation, might reduce missed opportunities to engage persons who use illicit opioids.

## Footnotes

* Fentanyl is a high-potency synthetic opioid that is up to 100 times more potent than morphine.

† Carfentanil is a fentanyl analog that is up to 100 times more potent than fentanyl; it is used to sedate large animals, such as elephants.

§ Clinical toxicology screening in emergency departments was limited to a qualitative urine immunoassay evaluation for amphetamines, barbiturates, benzodiazepines, cannabinoids, cocaine, methadone, opiates (e.g., codeine, morphine, and heroin), and phencyclidine. It did not confirm the presence of high-potency synthetic opioids, including fentanyl or fentanyl analogs. The case definition (which included substances from and symptoms attributable to nonopioid sedative drug classes) was employed to increase case-finding sensitivity in an outbreak setting lacking a surveillance system; specificity might have been improved by using a narrower case definition.

¶ A concurrent public safety investigation of this overdose outbreak was conducted by public safety agencies (law enforcement and fire department personnel) separate from the public health investigation; public safety investigators released their findings to public health investigators in March 2017, after legal proceedings concluded. Public safety investigation findings included comprehensive confirmatory opioid toxicology testing results of urine and blood specimens from patients who were subjects in the public health investigation.

** Mortality and drug testing data from Cabell County during the study period was not immediately available to public health investigators because of legal constraints. These data were reported to public health investigators separately in March 2017. Exact numbers were suppressed to preserve confidentiality in a small community.

†† Patient engagement was defined as program enrollment and receipt of formal addiction treatment.
